# Identification of Candidate Biomarkers for Idiopathic Thrombocytopenic Purpura by Bioinformatics Analysis of Microarray Data

**DOI:** 10.22037/ijpr.2020.113442.14305

**Published:** 2020

**Authors:** Samira Gilanchi, Hakimeh Zali, Mohammad Faranoush, Mostafa Rezaei Tavirani, Keyvan Shahriary, Mahyar Daskareh

**Affiliations:** a *Faculty of Paramedical Sciences, Shahid Beheshti University of Medical Sciences, Tehran, Iran. *; b *Proteomics Research Center, Faculty of Paramedical Sciences, Shahid Beheshti University of Medical Science, Tehran, Iran. *; c *School of Advanced Technologies in Medicine, Shahid Beheshti University of Medical Sciences, Tehran, Iran. *; d *Pediatric Growth and Development Research Center, Institute of Endocrinology, Iran University of Medical Sciences, Tehran, Iran. *; e *West Coast University PharmD campus, Los Angeles, Ca, USA. *; f *Department of Radiology, Ziyaian Hospital, Tehran University of Medical Sciences, Tehran, Iran.*

**Keywords:** Idiopathic Thrombocytopenic Purpura, ITP, Microarray, Gene expression, Biomarkers, Bioinformatics, System biology

## Abstract

Idiopathic Thrombocytopenic Purpura (ITP) is a multifactorial disease with decreased count of platelet that can lead to bruising and bleeding manifestations. This study was intended to identify critical genes associated with chronic ITP. The gene expression profile *GSE46922* was downloaded from the Gene Expression Omnibus database to recognize Differentially Expressed Genes (DEGs) by R software. Gene ontology and pathway analyses were performed by DAVID. The biological network was constructed using the Cytoscape. Molecular Complex Detection (MCODE) was applied for detecting module analysis. Transcription factors were identified by the PANTHER classification system database and the gene regulatory network was constructed by Cytoscape. One hundred thirty-two DEGs were screened from comparison newly diagnosed ITP than chronic ITP. Biological process analysis revealed that the DEGs were enriched in terms of positive regulation of autophagy and prohibiting apoptosis in the chronic phase. KEGG pathway analysis showed that the DEGs were enriched in the ErbB signaling pathway, mRNA surveillance pathway, Estrogen signaling pathway, and Notch signaling pathway. Additionally, the biological network was established, and five modules were extracted from the network. *ARRB1, VIM, SF1, BUB3, GRK5, *and *RHOG* were detected as hub genes that also belonged to the modules.* SF1* also was identified as a hub-TF gene. To sum up, microarray data analysis could perform a panel of genes that provides new clues for diagnosing chronic ITP.

## Introduction

Immune thrombocytopenic purpura (ITP) known as Idiopathic thrombocytopenic purpura is a multifactorial autoimmune bleeding disease associated with platelet destruction and discriminated by isolated thrombocytopenia (platelet count < 150,000 u/L) that was reported in almost 2 per 100,000 adults with a mean age of diagnosis of 50 years (1, 2). However, the vague pathogenesis, the abnormalities in the number and the function of different immune cells can play a crucial role in this disease. ITP phenotype, characterized by dysfunctional T-lymphocyte immunity, dysregulation in pre-B-cell, and T cell immunophenotypic markers, was recognized in bone marrow lymphocytes of pediatric ITP (3, 4). Besides, it is believed that membrane glycoproteins IIb-IIIa of platelet was targeted by immunoglobulin G autoantibody which is confirmed significantly by elevated CRP levels in ITP patients (5,6). These autoantibodies are recognized in 40–60% of patients and provide condition to Kupffer cells and splenic macrophages in the liver phagocytosis platelets (7). Other mechanisms include impaired production of platelet stimulatory hormone, thrombopoietin, reduced expression of human leukocyte antigen-G and immunoglobulin-like transcripts or secondary contributors such as childhood exposure to viruses, helicobacter pylori infection, and pregnancy (8-10). Zhang, *et al.,* determined six marker proteins that separate primary ITP from secondary ITP, including *NPS, EDN1, CORT, CLEC7A, CCL18*, and *NPPB*. Most of the detected proteins related to the immune system act as up/down-regulator in macrophages and platelet (11). Platelets can be recognized with the expression of CD38 as a prognostic marker for ITP (2).

As mentioned before, ITP classified as acute and chronic types and sub-categorized by primary and secondary etiology (9, 10). Besides, the alternative classification by international consensus guidelines organized 3 phases as newly diagnosed (up to 3 months), persistent (3-12 months’ duration), and chronic (over 12 months’ duration) (2, 6 and 11).

ITP patients were characterized by a decrease in platelet count of peripheral blood and variable bleeding symptoms. In severe cases, it may lead to fatal intracranial hemorrhage. Thus, prompt diagnosis and early therapeutic intervention are essential (12, 13).

There are no specific criteria for diagnosing ITP, and diagnosis is based on the exclusion criteria of the other diseases, such as lupus erythematosus, Von Willebrand disease type IIb, hemolytic uremic syndrome, Evans syndrome, disseminated intravascular coagulation, Posttransfusion purpura, paroxysmal nocturnal hemoglobinuria, myelodysplastic syndrome, lymphoproliferative disorders, Infections (viral, bacterial, parasitic), and drug-induced thrombocytopenia. Furthermore, antiplatelet antibody testing is not recommended because of high inter-laboratory variability and reduced sensitivity (14-16).

Microarray technology is a prevalent technique for studying the pattern of expression of a large number of genes to analyze a genome. Microarray data are important in many aspects of disease research, including primary research, target discovery, biomarker identification, and prognostic test determination. The methods used to analyze the data can have a profound effect on the interpretation of the results (17, 18). Network analysis of high-throughput data can be useful in breaking the gap between data production and drug targeting and helps to uncover biological complexity (19, 20). Therefore, to explore the molecular mechanism and discover specific biomarkers for chronic ITP compared with newly diagnosed in pediatrics, we applied bioinformatics techniques to analyze gene expression profiles of pediatric chronic ITP versus newly diagnosed and identify DEGs. For this aim, in the beginning, pediatric chronic ITP patients’ gene expression profiles were compared with pediatric newly diagnosed downloaded from GEO dataset. DEGs were identified using limma packages of the R software. The involvement of DEGs in the biological processes (BP), cellular components (CC), molecular functions (MF), and Kyoto Encyclopedia of Genes and Genomes (KEGG) were assessed with DAVID online tool. DEGs visualized using Cytoscape software. We applied the network analysis using Cytoscape to predict probable biomarkers. The panther database was used for transcriptional regulatory network construction. These studies could help find crucial genes that might be applied for appropriate diagnostics and treatment strategies in ITP.

## Experimental

*Microarrays data*

The Gene Expression Omnibus (GEO, http://www.ncbi.nlm.nih.gov/geo) is a public dataset for storage microarray, and next-generation sequencing data is freely available for users. In this study, ITP genomic data were obtained from GEO with the series accession number GSE46922 and the platform GPL570 (Affymetrix Human Genome U133 Plus 2.0 Array). This dataset included data from thirteen blood samples: seven newly diagnosed and six chronic samples described by Margareta Jernas *et al.* (21).

*Data processing*

Data retrieved from GEO were analyzed with R software to discover significantly expressed genes by employing various statistical tests, mainly, the t-statistics and *P*-value. R software is a fascinating tool to discriminate two or more groups of samples to classify genes, differentially regulated following the same experimental condition. This software can estimate the *P*-value for significant outcomes by utilizing Limma R packages from the Bioconductor project. Benjamini false discovery rate was concerned in this outline. Here the genes were chosen for more evaluation with *P*-value < 0.05, and-0.5 >M > 0.5 (M is log2 fold change).

*Functional and pathway enrichment analysis*

Up-regulated and down-regulated genes were analyzed separately by the DAVID enrichment database (version 6.8) (https://david.ncifcrf.gov). The Database for Annotation, Visualization, and Integrated Discovery (DAVID) is a web-accessible program that provides a comprehensive set of functional annotation tools to disclose the biological meaning behind gene sets. DAVID contains numerous public sources of protein and gene annotation from more than 65,000 species (22). Gene Ontology and KEGG pathway analysis were performed using the DAVID database for functional analysis of the gene lists. We used the functional annotation clustering; to reveal the clusters enriched in gene ontology and KEGG pathway terms with the enrichment score number. Gene Ontology (GO; www.geneontology.org) and the Kyoto Encyclopedia of Genes and Genomes (KEGG; www.genome.ad.jp/KEGG) enrichment analysis were performed to identify DEGs. GO was used for categorization, including biological process, molecular function, and cellular component, which is widely used in bioinformatics and increases the possibility of indentifying the most correlative mechanisms. KEGG was used for understanding the most relevant pathway of informative genes. 

*Network construction and modules selection*

DEGs interaction network can clarify the molecular mechanism of cellular processing. Functional interaction between DEGs was constructed with Cytoscape (version 3.5.1). In this study, the network was extended with the Cytoscape public database. Highly connected nodes were selected as hubs. Some nodes with the highest betweenness centrality were nominated as bottleneck nodes. Then, Molecular Complex Detection (MCODE) was used for screening modules. The functional enrichment analysis of DEGs in each module was performed by DAVID.

*Transcriptional regulatory network construction*

In order to identify the transcription factor (TF) nodes in the network, the PANTHER Classification System database (http://www.pantherdb.org/) was used (23). Then the transcriptional regulatory network was visualized by Cytoscape. 

## Results

*Data screening*

**Figure 1:**
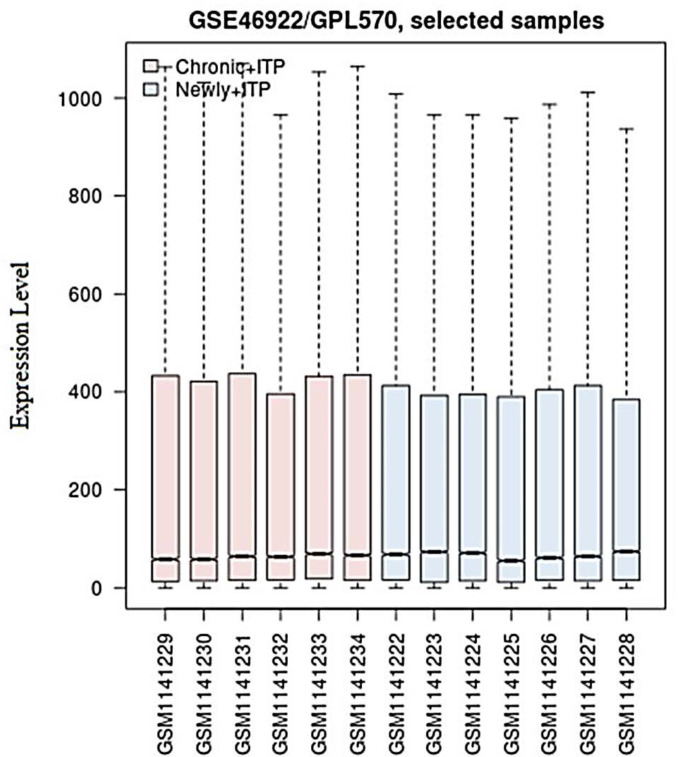
Box plot of expression data by analyzing GSE46922 that contain seven newly diagnosed ITP and six chronic ITP samples

Based on *P*-value < 0.05 in comparison to newly diagnosed ITP/chronic ITP, 132 DEGs were identified, consisting of 78 up-regulated (Supplementary Table S1) and 54 down-regulated genes (Supplementary Table S2). As shown in (Figure 1), the medians located at the same level after performing data normalization with R software, indicating a perfect effect. 

*Gene ontology and pathway enrichment analysis*

Gene Ontology and Pathway functional enrichment analysis were performed according to the *P*-values < 0.05 on the identified DEG. The enriched term of BP for up-regulated genes was reported in (Table 1 and Supplementary Table S3). They were significantly involved in 10 significant clusters of biological processes, associated with regulation of autophagy, cell cycle checkpoint, regulation of gene expression, cellular component organization or biogenesis, positive regulation of cell projection organization, macromolecule metabolic process, sister chromatid segregation, the establishment of protein localization to the membrane, and regulation of protein tyrosine kinase activity. The up-regulated genes were located in 5 clusters associated with the intracellular part, organelle, nucleus, intracellular non-membrane-bounded organelle, chromosome, centromeric region, and ciliary membrane (Supplementary Table S4). Significant Molecular function represented in (Supplementary Table S5) involved 2 cluster link to nucleic acid binding and protein kinase activity.  

Moreover, the gene ontology related to biological process terms were over-represented in down-regulated DEGs with significant *P*-value which mainly involved in the modification of morphology or physiology of other organism involved in symbiotic interaction, cellular response to monosaccharide stimulus, programmed cell death, histone methylation, negative regulation of sequence-specific DNA binding transcription factor activity, and positive regulation of binding (Table 2 and Supplementary Table S6). 

The significant cellular components, related to down-regulated genes contained four clusters that were mainly involved in intracellular, organelle part, membrane-bounded organelle, lysosomal membrane, lytic vacuole membrane, and chromosomal part. (Supplementary Table S7). Two significant molecular function clusters for down-regulated genes, represented in (Supplementary Table S8), were related to lipase activity, hydrolase activity, acting on ester bonds, and structure-specific DNA binding.

The significant pathway represented in (Table 3) for up-regulated and down-regulated DEGs .the pathway enrichment analysis for up-regulated DEGs indicated these genes involved in the ErbB signaling pathway, mRNA surveillance pathway, and the Estrogen signaling pathway. Whereas, only the Notch signaling pathway is related to down-regulated genes. 

*Network construction and modules selection*

**Figure 2:**
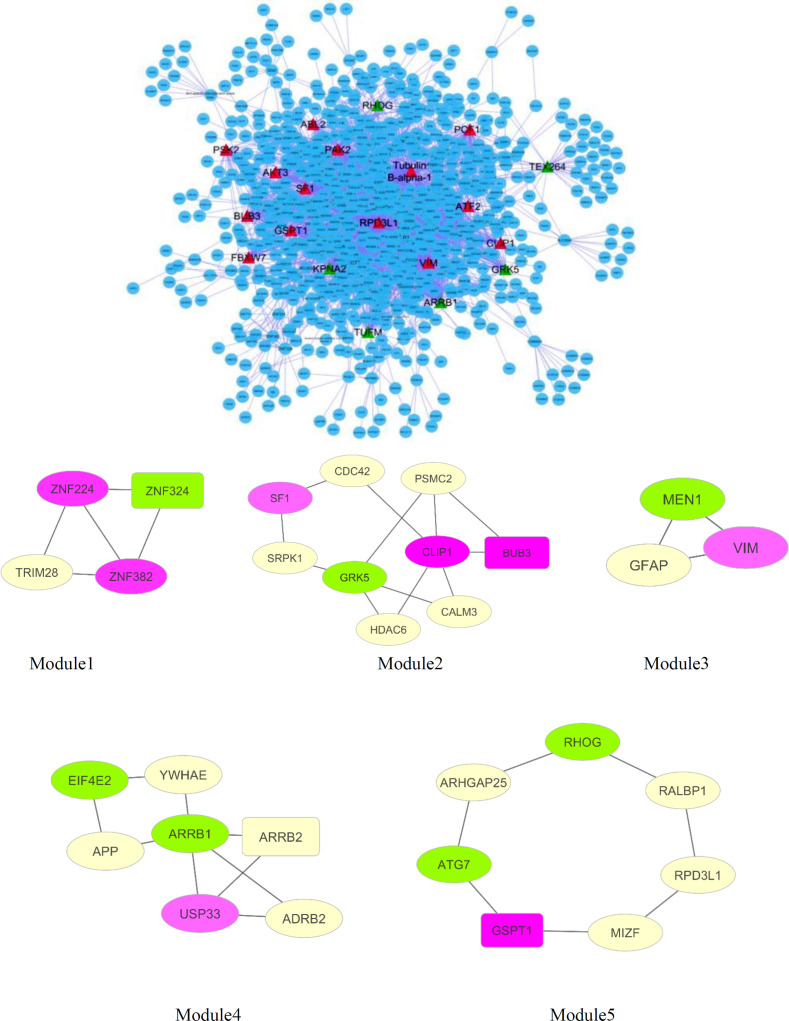
(A) network with 1137 nodes and 2647 edges. Unregulated hub genes were shown with red triangle nodes while down-regulated represented with green color (B) Significant modules selected from the network. Pink modules illustrated up-regulated genes, while green nodes illustrated down-regulated genes. Seed nodes are shown in rectangular shape

Based on public databases existing on Cytoscape, the PPI network of DEGs was established. Network analysis was shown consisting of 1137 nodes and 2647 edges (Figure.2A). The cut-off criterion of hub gene selection was set at ≥ 40 degrees. Based on this cut-off, twenty hubs are recognized in the network. Therefore, regarding the cut-off criteria, seventeen genes of DEGs were selected as hub nodes. They consisted of eleven up-regulated (*ATF2, VIM, PAK2, SF1, BUB3, PCF11, PCF12, FBXW7, GSPT1, CLIP1, ABL2*) and six down-regulated (*ARRB1, KPNA2, GRK5, TUFM, RHOG, TEX264*) genes (Table 4). 

 Twenty of the highest betweenness centrality including fifteen genes of DEGs were selected as bottleneck containing nine up-regulated (*BBS2, RPRD1A, FNBP4, TUBE1, ABCA5, EIF4E3, TNRC6A, TBC1D5, VIM*) and six down-regulated (*TMEM214, COPRS, MAU2, MRPL45, TBC1D9B, ARRB1*) genes (Table 5). Two of the mentioned genes,including VIM and ARRB1, appeared among hub nodes. These two genes were dentified as hub-bottleneck genes. Which confirms the important role of these two genes.

The functional modules were assessed using the MCODE plugin. Five modules were identified including thirty nodes and 36 edges that comprised Module 1 (*ZNF324, ZNF224, ZNF382, TRIM28*), module 2 (*BUB3, CDC42, GRK5, PSMC2, SF1, HDAC6, SRPK1, CLIP1*), module 3 (*VIM, MEN1, GFAP*), module 4 (*APP, EIF4E2, ARRB1, ARRB2, ADRB2, YWHAE, USP33*), module 5 (*ATG7, GSPT1, RALBP1, MIZF, RPD3L1, ARHGAP25, RHOG*) (Figure 2B).

We found six DEGs existing in both hub genes and modules, which have significant* P*-value (*P*-value < 0.05) for enriched BP. These genes included three up-regulated genes and three down-regulated genes (Table 6). 

*Transcriptional regulatory network construction:*

One hundred twenty-one nodes with TF function have been identified from 1137 network nodes using the panther database. By using Cytoscape, these 121 nodes have been visualized in a regulatory network.

Five genes of thirty genes existing in modules are TF, including *ZNF224, ZNF382, TRIM28, MIZF*, and *SF1*. *ZNF382, TRIM28, ZNF224* belong to module one, *SF1* belongs to module two, and *MIZF *belongs to module 5.

Further analysis of potentially remarkable modules was performed by detecting TFs with a high degree of connections with other nodes, the so-called hub-TFs.

**Figure 3:**
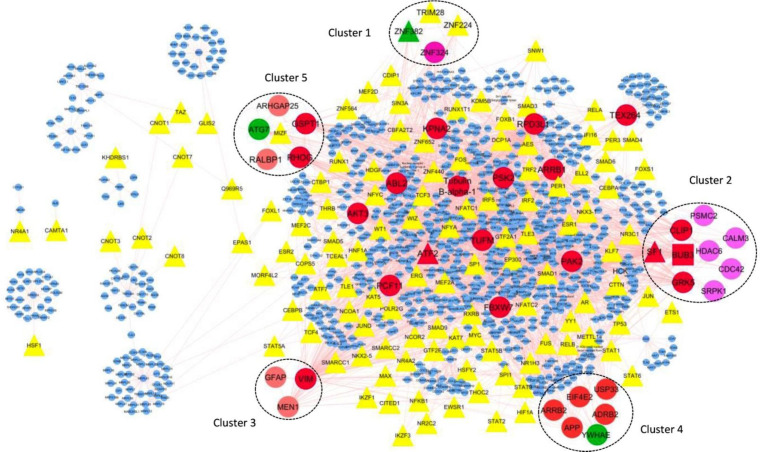
Visualization in Cytoscape of interactions between TFs, modules and hub-TFs. TFs are shown as triangle. Hubs are displayed in red ellipses. Modules showed by number with different color that contains the nodes are hub (red node), seed (green node) and TF (yellow and red triangle). There is just one red rectangle in module No.2 related to the node that is hub-seed gene. Red triangle related to the nodes are hub-TFs and green triangle is a node related seed-TFs. SF1 and ATF2 are hub-TFs and ZNF382 is a seed-TF. ZNF382 and SF1 are the members of modules No.1 and No.2 respectively

It should be noted that SF1 is the hub node in the transcriptional regulatory network. *SF1*, as a TF encoding gene is a hub-TF (Figure.3). 

## Discussion

Diminished platelet production and enhanced platelet destruction are the familiar characters of ITP (24). However, the first hit for dysregulation of the immune system in ITP remains unknown (25). Understanding the molecular and physiopathological mechanisms of ITP requires many efforts to design new preventive and therapeutic strategies. Due to the interaction of genes and environmental factors in common human diseases, a more integrated biological approach is needed to solve these complexities (26). DNA microarrays are used as a powerful technique in biomedical research. This method has attracted much attention from scientists because of its ability to identify thousands of genes and even the entire genome simultaneously (26). Systemic network analysis of high-throughput data is the most useful technique to explain the important implications of life science. Network features, such as composition and topology are highly relevant to vital cellular functions, so they are critical in biological science research (27). This study tries to find essential genes and mechanisms by bioinformatics analysis of GSE46922 microarray data, which are different between the newly diagnosed and the chronic ITP. This study identifies, 131 DEGs, consisting of 78 up-regulated genes and 53 down-regulated genes, which are differentially expressed between, the newly diagnosed ITP and chronic ITP-.

Our enrichment analysis of the up-regulated DEGs showed that autophagy played a significant role in ITP. There is evidence that the positive regulation of autophagy is the most relevant biological process in ITP associated with the expressed genes in the chronic phase. Autophagy induces to the maintenance of platelet life and physiological functions (28). Improper expression of molecules in the autophagy pathway has been also determined in ITP patients lymphocytes (29). Elevating platelet autophagy has been also shown to diminish platelet destruction by prohibiting apoptosis and amending platelet viability (28). Besides, particular evidence implied that megakaryocytes undergo autophagy in ITP patients (30). The apoptotic process was diminished in accordance with activate autophagy process in chronic ITP. 

Our study has shown that down-regulated genes in the chronic phase were mainly enriched in the Notch signaling, closely related to hematopoiesis, which involves the evolving hematopoietic system to generate hematopoietic stem cells and the development of immune cells like in T-cells or progress several autoimmune diseases like ITP (32). Rania Mohsen Gawdat *et al*. found the correlation of *Notch1/Hes1* gene expression levels in Egyptian paediatric patients with newly diagnosed and persistent primary ITP (31, 32). We detected this pathway in newly diagnosed ITP while down-regulated in the chronic phase, and this data has shown that the Notch pathway is replaced by the *ErbB* signaling pathway, mRNA surveillance pathway, and Estrogen signaling pathway over time to display the chronic phase symptom. Also molecular crosstalk among Notch signaling pthway with *ErbB* and Estrogen signaling pathways was acknowledged in breast cancer (33). This study also confirms the crosstalk between emerging *ErbB* and Estrogen pathway and inhibition of the Notch signaling pathway in ITP. The mRNA surveillance pathway was enriched by the up-regulated genes related to the quality control mechanism that targets aberrant mRNAs for degradation (34). This pathway was not reported for ITP but confirm this mechanism in autoimmune disease and cellular defense against virus invasion. Mutations affecting the mRNA surveillance machinery cause chronic activation of defense programs, resulting in autoimmune phenotypes. The Systemic lupus erythematosus (SLE) as a human autoinflammatory and autoimmune disorders are notably linked to this system deviation (34). ITP manifests several symptoms of mimicking diseases like SLE; therefore, one might be aware of this similarity emphasizing with several investigations. Besides, this pathway enriched from down-regulated genes in the chronic phase; it implies that the chronic phase of ITP can be due to perturbations in the pathways.

The network analysis also demonstrated that there are interactions among the DEGs. 

Our network analysis revealed a set of candidate genes (three up-regulated and three down-regulated) for the investigation of biomarkers or molecular mechanisms of ITP, which was significantly correlated with chronic ITP, including *BUB3, GRK5, SF1, VIM, ARRB1, *and* RHOG*. 

Our network analysis also verifies the Notch signaling pathway in ITP. In this study, *ARRB1* was considered a hub-bottleneck protein with a high degree and high betweenness centrality value. This protein is strongly related to the Notch signaling pathway. Due to its unique features, it has an attractive advantage for drug targeting. 

One of the essential genes that play an indispensable role in the maturation of hematopoietic precursors is *Vimentin* (*VIM*) that belongs to hub-bottleneck protein. Alteration in expression of *VIM* has been recognized in the maturation process of the megakaryocytic, granulomonocytic, erythroid, and lymphoid lineages (35). Up-regulated *VIM* has been also shown in the formation of fully active macrophage-like cells and macrophage polykaryons (36). *Rho GTPases* (*RhoG*) is one of the crucial members of our analysis, which has a central regulatory role in platelet production and megakaryocyte maturation (37).

One of the most important genes in this research was *SF1*. In addition to being a hub, integrating TF’s expression data into Cytoscape indicated that *SF1 *is also a TF. Kenichi Yoshida *et al*. reported that there is a mutation in *SF1* in hematologic malignancies, but its frequency was not at confidence level for presentation to clinical associations (38).

The G-protein-coupled receptor kinase 5 (GRK5) is a critical member of the threonine/serine kinase family that phosphorylates and regulates the G-protein-coupled receptor (GPCR) signaling pathway. GRK5 has a key role in several diseases; for example, GRK5 is a decisive pathogenic factor in early Alzheimer’s disease, hepatic steatosis and metabolic disorders such as type II diabetes and obesity, injured and failing heart and cancer (39-44). GRK*5* also has multiple roles in *TLR *(Toll-Like Receptor) signaling, which were described as a family of receptors involved in recognizing pathogen-associated molecular patterns (PAMPs) derived from microbes. Moreover, the importance of *TLRs* has been identified in several inflammatory diseases, including non-infectious diseases (45, 46). In addition, detection of GRK5 expression provides a target for determining the effectiveness of drugs and determining patient prognosis in cancer (47).

The *BUB3* is one of the mitotic checkpoint proteins specified by a group of evolutionarily conserved genes. It is believed that the failure of the *BUB* gene family as a surveillance system is a critical components of the regulatory process which causes genomic instability. This gene family encodes proteins that are a part of a large multi-protein kinetochore complex (48, 49). The *BUB3*’s importance was found in colorectal cancer at a young age and in low-grade breast cancers (50, 51).

The use of omics technology to identify the mechanism of disease and the discovery of biomarkers has received much attention in recent years. Microarray and proteomics approaches can help to solve biological complexities by creating an extensive list of expressed transcripts that are simultaneously (52). As mentioned in the introduction, Zheng and his colleagues were able to introduce six important markers for the diagnosis of ITP by using Proteomics technology in 2016 (11). However, they have not yet been used in the clinic. Our study using microarray data analysis introduces six new markers that can clarify the pathogenesis of the ITP and need many examinations for clinic application.

**Table 1:** Gene ontology enrichment analysis based on biological analysis of up-regulated DEGs. They were selected with significant value *P* < 0.05. Enrichment Score is related the type of analysis in the DAVID database selecting the"functional annotation clustering" for analysis of gene lists

**Category**	**Term**		***P*** ***-*** **Value**
**Annotation Cluster 1**	**Enrichment Score: 1.6089173346492356**		
GO:1902589	single-organism organelle organization		0.005313
GO:0000226	microtubule cytoskeleton organization		0.020728
GO:0007017	microtubule-based process		0.026109
**Annotation Cluster 2**	**Enrichment Score: 1.5454786370206883**		
GO:0010506	regulation of autophagy		0.003125
GO:0010508	positive regulation of autophagy		0.03688
**Annotation Cluster 3**	**Enrichment Score: 1.1276637957883262**		
GO:0000075	cell cycle checkpoint		0.010137
GO:0022402	cell cycle process		0.010174
GO:0000077	DNA damage checkpoint		0.017372
GO:0007049	cell cycle		0.017944
GO:0031570	DNA integrity checkpoint		0.020624
GO:0007093	mitotic cell cycle checkpoint		0.020967
GO:0045930	negative regulation of mitotic cell cycle		0.047204
**Annotation Cluster 4**	**Enrichment Score: 1.0524732568167308**		
GO:0016043	cellular component organization		0.03475
GO:0006996	organelle organization		0.046543
GO:0071840	cellular component organization or biogenesis		0.04844
**Annotation Cluster 5**	**Enrichment Score: 0.9254147029064898**		
GO:0031344	regulation of cell projection organization		0.004985
GO:0010975	regulation of neuron projection development		0.018065
GO:0030030	cell projection organization		0.020064
GO:0031346	positive regulation of cell projection organization		0.02586
GO:0031175	neuron projection development		0.030331
GO:0030182	neuron differentiation		0.035135
**Annotation Cluster 6**	**Enrichment Score: 0.9097209940740182**		
GO:0043170	macromolecule metabolic process		0.001037
GO:0010468	regulation of gene expression		0.008941
GO:0060255	regulation of macromolecule metabolic process		0.008953
GO:0019222	regulation of metabolic process		0.011749
GO:0044260	cellular macromolecule metabolic process		0.014544
GO:0010467	gene expression		0.017106
GO:0010558	negative regulation of macromolecule biosynthetic process		0.033922
GO:0009892	negative regulation of metabolic process		0.038268
GO:0010605	negative regulation of macromolecule metabolic process		0.041577
GO:0031327	negative regulation of cellular biosynthetic process		0.043543
GO:0031324	negative regulation of cellular metabolic process		0.044105
GO:0009890	negative regulation of biosynthetic process		0.047731
GO:0051172	negative regulation of nitrogen compound metabolic process		0.048092
**Annotation Cluster 8**	**Enrichment Score: 0.7138986023041735**		
GO:0007062	sister chromatid cohesion		0.011798
GO:0000819	sister chromatid segregation		0.049886
**Annotation Cluster 11**	**Enrichment Score: 0.5802981069084664**		
GO:0090150	establishment of protein localization to membrane		0.046903
**Annotation Cluster 17**	**Enrichment Score: 0.42916203800540753**		
GO:0018108	peptidyl-tyrosine phosphorylation		0.009263
GO:0018212	peptidyl-tyrosine modification		0.00948
**Annotation Cluster 18**	**Enrichment Score: 0.40498640863411367**		
GO:0018108	peptidyl-tyrosine phosphorylation		0.009263
GO:0018212	peptidyl-tyrosine modification		0.00948
GO:0044260	cellular macromolecule metabolic process		0.014544
GO:0061097	regulation of protein tyrosine kinase activity		0.023351

**Table 2:** Gene ontology enrichment analysis based on biological analysis of down-regulated DEGs. They were selected with a significant value *P* < 0.05. Enrichment Score is related the type of analysis in the DAVID database, selecting the “functional annotation clustering” for analysis of gene lists

**Category**	**Term**	***P*** **-** **Value**
**Annotation Cluster 2**	**Enrichment Score: 0.7613112520752194**	
GO:0044267	cellular protein metabolic process	0.011896
GO:0043412	macromolecule modification	0.01846
GO:0019538	protein metabolic process	0.018791
GO:0006807	nitrogen compound metabolic process	0.019514
GO:0009059	macromolecule biosynthetic process	0.021932
GO:0043170	macromolecule metabolic process	0.028871
GO:0044249	cellular biosynthetic process	0.033903
GO:0034641	cellular nitrogen compound metabolic process	0.034934
GO:0044237	cellular metabolic process	0.043685
GO:0008152	metabolic process	0.044952
GO:0044238	primary metabolic process	0.04675
GO:0006464	cellular protein modification process	0.047376
GO:0036211	protein modification process	0.047376
GO:0009058	biosynthetic process	0.048575
GO:0071704	organic substance metabolic process	0.04992
**Annotation Cluster 3**	**Enrichment Score: 0.7085147802867421**	
GO:0051817	modification of morphology or physiology of other organism involved in symbiotic interaction	0.023652
GO:0035821	modification of morphology or physiology of other organism	0.033336
**Annotation Cluster 5**	**Enrichment Score: 0.5438770693729118**	
GO:0016570	histone modification	0.005122
GO:0016569	covalent chromatin modification	0.012772
GO:0006325	chromatin organization	0.012912
GO:0090630	activation of GTPase activity	0.017421
GO:0032092	positive regulation of protein binding	0.017839
GO:0006996	organelle organization	0.019368
GO:0018205	peptidyl-lysine modification	0.01994
GO:0051817	modification of morphology or physiology of other organism involved in symbiotic interaction	0.023652
GO:0071333	cellular response to glucose stimulus	0.026068
GO:0071331	cellular response to hexose stimulus	0.027564
GO:0071326	cellular response to monosaccharide stimulus	0.027564
GO:0051276	chromosome organization	0.029444
GO:0006915	apoptotic process	0.030391
GO:0071322	cellular response to carbohydrate stimulus	0.032253
GO:0035821	modification of morphology or physiology of other organism	0.033336
GO:0001678	cellular glucose homeostasis	0.034432
GO:0012501	programmed cell death	0.042073
GO:0016571	histone methylation	0.0443
GO:0006464	cellular protein modification process	0.047376
GO:0036211	protein modification process	0.047376
GO:0043433	negative regulation of sequence-specific DNA binding transcription factor activity	0.048006
GO:0051099	positive regulation of binding	0.0499

**Table 3:** KEGG Pathway enrichment analysis of up-regulated and down-regulated DEGs. They were selected with a significant value *P* < 0.05 in the DAVID database

	**Term**	**Description**	***P*** **-value**	**Genes**
**Up-regulated DEGs**				
1	hsa04012	ErbB signaling pathway	0.033682	PAK2, ABL2, AKT3
2	hsa03015	mRNA surveillance pathway	0.036574	PCF11, GSPT1, MSI2
3	hsa04915	Estrogen signaling pathway	0.042636	FKBP5, AKT3, ATF2
**Down-regulated DEGs**				
1	hsa04330	Notch signaling pathway	0.009201	HDAC1;MFNG;DTX1

**Table 4:** Hub gene with the cut-off criterion degrees ≥ 40. Three genes did not excist in DEGs and were added by Cytoscape software. Bottleneck genes showed by star in the betweenness centrality column

	**UniProtKB ID**	**Gene name**	**Degree**	**Betweeness centrality**
**Up-regulated**				
1	P15336	ATF2	124	0.095148
2	P08670	VIM	111	0.123853*
3	Q13177	PAK2	101	0.07973
4	Q15637	SF1	90	0.084552
5	O43684	BUB3	85	0.058294
6	O94913	PCF11	77	0.037986
7	Q9Y243	PCF12	62	0.03668
8	Q969H0	FBXW7	53	0.025345
9	P15170	GSPT1	49	0.037137
10	P30622	CLIP1	46	0.012378
11	P42684	ABL2	42	0.023944
**Down-regulated**				
1	P49407	ARRB1	125	0.121072*
2	P52292	KPNA2	81	0.066201
3	P34947	GRK5	59	0.036356
4	P49411	TUFM	56	0.035678
5	P84095	RHOG	40	0.028479
6	Q9Y6I9	TEX264	40	0.055543
**Added by network**				
1	Q13547	RPD3L1	202	0.236783*
2	Q71U36	Tubulin B-alpha-1	134	0.135022*
3	Q7L7X3	PSK2	42	0.017018

**Table 5:** The genes with the highest betweenness centrality were selected as the bottleneck. Five genes did not excisted among the DEGs and were added by cytoscape software. Stars in degree column show the importance of these gnes as hub genes

	**UniProtKB ID**	**Gene name**	**Betweenness centrality**	**Degree**
**Up-regulated**				
1	Q9BXC9	BBS2	1	27
2	Q96P16	RPRD1A	1	24
3	Q8N3X1	FNBP4	1	6
4	Q9UJT0	TUBE1	1	2
5	Q8WWZ7	ABCA5	1	2
6	Q8N5X7	EIF4E3	1	2
7	Q8NDV7	TNRC6A	0.44370861	27
8	Q92609	TBC1D5	0.48979592	11
9	P08670	VIM	0.41453744	111*
**Down-regulated**				
1	Q6NUQ4	TMEM214	1	13
2	Q9NQ92	COPRS	1	8
3	Q9Y6X3	MAU2	1	5
4	A0A087X2D5	MRPL45	0.807327	37
5	Q66K14	TBC1D9B	0.76268116	14
6	P49407	ARRB1	0.12107173	125*
**Added by network**				
1	Q13618	CUL3	0.48846676	2
2	Q13547	RPD3L1	0.23678326	202*
3	Q9H492	MAP1LC3A	0.23550725	2
4	Q9H0R8	GABARAPL1	0.23550725	2
5	Q71U36	Tubulin B-alpha-1	0.13502234	134*

**Table 6:** Key genes related to chronic ITP that selected based on multiple criteria of data analysis. Hub gene with the cut-off criterion degrees ≥ 40 which are also existed in modules selected as potential biomarkers for chronic ITP. The fold change in expressed genes in microarray selected based on M index that is log2 fold change

	**Gene name**	**Gene ID**	**Degree**	**Betweeness centrality**	**M**	**Biological process**
**Up-regulated**						
1	VIM	P08670	111	0.12385346	1.077912156	positive regulation of protein ubiquitination involved in ubiquitin-dependent protein catabolic process (GO:2000060)
2	SF1	Q15637	90	0.08455183	2.419055031	mRNA splice site selection (GO:0006376),spliceosomal complex assembly (GO:0000245),mRNA 3'-splice site recognition (GO:0000389)
3	BUB3	O43684	85	0.05829374	0.846438817	regulation of translation (GO:0006417)
**Down-regulated**						
1	ARRB1	P49407	125	0.12107173	-1.855926621	regulation of Notch signaling pathway (GO:0008593), negative regulation of sequence-specific DNA binding transcription factor activity (GO:0043433), negative regulation of NF-kappaB transcription factor activity (GO:0032088), positive regulation of histone H4 acetylation (GO:0090240), desensitization of G-protein coupled receptor protein signaling pathway (GO:0002029), regulation of histone H4 acetylation (GO:0090239), positive regulation of cellular metabolic process (GO:0031325), contractile actin filament bundle assembly (GO:0030038), stress fiber assembly (GO:0043149), negative regulation of cytokine production (GO:0001818), positive regulation of peptidyl-lysine acetylation (GO:2000758), negative regulation of interleukin-8 production (GO:0032717), modification-dependent protein catabolic process (GO:0019941)
2	GRK5	P34947	59	0.03635575	-0.896145434	tachykinin receptor signaling pathway (GO:0007217), regulation of signal transduction (GO:0009966), positive regulation of cell proliferation (GO:0008284), regulation of cell proliferation (GO:0042127)
3	RHOG	P84095	40	0.02847864	-1.37279767	Rac protein signal transduction (GO:0016601), activation of GTPase activity (GO:0090630), positive regulation of GTPase activity (GO:0043547), positive regulation of cell proliferation (GO:0008284), engulfment of apoptotic cell (GO:0043652), phagocytosis, engulfment (GO:0006911), neutrophil degranulation (GO:0043312),neutrophil activation involved in immune response (GO:0002283), neutrophil mediated immunity (GO:0002446), regulation of cell proliferation (GO:0042127)

## Conclusion

The current study has obtained DEGs using comprehensive bioinformatics analysis of high-throughput data released from microarray analysis to find the possible biomarkers. In summary, a total of 132 DEGs were screened, and six genes, including *BUB3, GRK5, SF1, VIM, ARRB1*, and *RHOG*, previously have not been reported as signature genes in ITP; here we found that they might play critical roles in chronic ITP. This research contributes new insights into the molecular mechanisms of newly diagnosed ITP and chronic ITP. These six genes together could be considered as a panel of biomarkers to differentiate newly from chronic ITP. Thus, additional investigations are needed to focus on the clinical application of these genes.
